# Characterization of the remediation of chromium ion contamination with bentonite by terahertz time-domain spectroscopy

**DOI:** 10.1038/s41598-022-15182-x

**Published:** 2022-07-01

**Authors:** Hang Cheng, Hao-chong Huang, Meng-fan Yang, Mei-hui Yang, He Yan, Spozmai Panezai, Zhi-Yuan Zheng, Zhuo Zhang, Zi-li Zhang

**Affiliations:** 1 School of Science, China University of Geosciences (Beijing), Beijing, 100083 China; 2 School of Earth Sciences and Resources, China University of Geosciences (Beijing), Beijing, 100083 China; 3 Institute of Physics, Faculty of Physics, Astronomy and Informatics, Nicolaus Copernicus University in Toruń, ul. Grudziądzka 5, 87-100 Toruń, Poland; 4 School of Land Science and Technology, China University of Geosciences (Beijing), Beijing, 100083 China

**Keywords:** Optics and photonics, Materials science, Techniques and instrumentation

## Abstract

Heavy metal pollution of agricultural and urban soils limits economic progress in the rapidly developing society. Terahertz technology is applied to detect heavy metal pollutants under existence of multiple pathways of their dissemination. In this study, terahertz time-domain spectroscopy (THz-TDS) is employed as an advanced probing technique in combination with traditional detecting methods to measure the adsorption ability of trivalent chromium ions on bentonite. The concentration of chromium ions and the weight of bentonite are known to influence on the adsorption capacity of the latter. It is tested here by both qualitative and quantitative measurements of two mentioned parameters. The adsorption process of chromium ions by bentonite is monitored using THz-TDS. The adsorptions signal from samples at 0.5 THz gradually increases with the increase of bentonite weight or chromium ion concentration. It would appear to indicate that terahertz could be used for quantitative detection of metal ions. Secondly, the ratios of results obtained by inductively coupled plasma mass spectrometry (ICP-MS) and the THz-TDS ones are stabilized at 0.105  ±  0.014 as the bentonite weight or chromium ion concentration increase. Such finding confirms that terahertz technology can be used for the quantitative detection of metal ions. Using the relationship between the ICP-MS test results and the THz-TDS ones, the amplitude value of bentonite is obtained to be 13.925 at the concentration of chromium ions of 0.05 mol/L, the mass of bentonite sample involved in adsorption of 1.5 g, and the detection frequency in THz-TDS measurements of 0.5 THz. The adsorption coefficient of bentonite is calculated to be 1.44%. Increase of the chromium ion concentration to 0.2 mol/L, and the mass of bentonite involved in adsorption to 3 g leads to the increase of the amplitude corresponding to adsorbed chromium ions to about 19.463, and the adsorption coefficient to about 2.1%. Obtained results demonstrate that terahertz technology is promising to meet the ever-increasing requirements in mineral analyses for rapid detection of chemical contaminants and measurement of the adsorption efficiencies of materials.

## Introduction

Rapid industrialization has caused a large number of issues resulted from pollution, which must not be ignored. Environmental deterioration has become a national concern. In particular, heavy metals are among the contaminants that are the most widely discussed at the national and international levels. Pollution with heavy metals causes soil poisoning^1^. Many heavy-metal ions come from paper-making, alloy production, manufacturing and other industries^2^. Therefore, environmental legislation is gradually becoming more rigid^3^ and the detection of pollutants has become one of the mainstream research directions.

Chromium (Cr) is widely applied in industry, such as in tanning, electroplating, ore refining, wood preservation and pigmentation^4,5^, which inevitably results in the discharge of Cr-containing industrial effluents. Chromium is considered as one of the top 20 contaminants in the Superfund priority list of hazardous substances for the past 15 years^6^. Due to the hazardous nature of Cr pollution, it is necessary to elaborate practical methods for the remediation of Cr-contaminated soil and water. Cr(III) is naturally present in the environment, whereas it is generally removed by tertiary treatment of wastewater or by advanced physical/chemical treatments, which constitute the processes of filtration. Among numerous methods, the best-established one is the removal of heavy metals by adsorption^7^. Characterized by high metal binding capacities, low cost, varying pH range, easy operation, flexibility, and simple design, adsorption can be successfully implemented in the pollution management^8^. Based on the previous studies, lichen, Celtek clay, moss, red algae, olive leaves, vermiculite and amino acid residues are proposed to use as adsorbents. The biosorption of chromium ions by chemical ion exchange was observed for lichen^9^. Optimum biosorption conditions in terms of biomass dosage, contact time and temperature were determined. Chromium ions were also trapped by physical adsorption on Celtek clay^10^. At this, the adsorbent dosage, shaking time and temperature affected the sorption results. The biosorption of Cd(II) and Cr(III) on moss (*Hylocomium splendens*) took place by chemical ion exchange^11^. Similar biosorption processes of chromium were observed for red algae (*Ceramium virgatum*) and olive leaf powder^12,13^. Unlike moss, the adsorption on vermiculite was essentially physical^14^. Amino acid residues are unique among all the materials for heavy metal trapping. In particular, they absorb Cr(III) without being damaged as organic materials^15^. All of the substances described above followed well pseudo-second-order adsorption kinetics.

Bentonite, which is one of the main components of soil, is often used as the experimental object in the study of heavy metal adsorption. It is a traditional low-cost efficient adsorbent, which has a high potential for application in removing heavy metals from wastewater due to its abundance, chemical and mechanical stability, high adsorption capability and unique structural properties^16,17^. Bentonite is a natural mineral with a layered hydrated aluminosilicate structure^18,19^. It contains montmorillonite, which is a swollen clay mineral with a 2:1 sheet structure, different from kaolinite of clay minerals. Montmorillonite has good ion exchange properties^20^.

Modified-bentonite family substances demonstrate stronger adsorption as compared to that of already discussed materials. Chemical and physical modification of bentonite can be done by various methods. Bentonite (BNT)–Gum Arabic (GA) composite excellently removes Th(IV) ions from aqueous media, and the adsorption mechanism fits perfectly to the pseudo-second-order (PSO) model^21^. Bentonite was also modified by ethylene diamine (EDA)-trimesoyl chloride (TMC) polymer to create a novel adsorbent for efficient removal of rhodamine B dye (RB) from wastewater. The adsorption process by this adsorbent also follows the pseudo-second-order kinetic model^22^.

The reasons to use bentonite as natural adsorbent can be explained as follows. First, cations such as K, Cu, Mg, Na, etc. are mainly found in montmorillonite inter-layers^23^. These cations and crystal structures are unstable, which leads to ion-exchange reactions^24^. Second, high-valence metal cations are easier attracted to clay particles than monovalent ones, which is a suitable reason to use chromium ions for experiments. Third, montmorillonite attracts more Cr(III) ions than kaolin^25^. Finally, a significant role in the absorption by bentonite is played by cation exchange reactions^26^ and sponge structure of this material^27^. The temperature has an influence on the adsorption by bentonite, namely the adsorption capacity decreases with the increase of temperature due to the spontaneous and exothermic nature of adsorption process^28^.

Next, chromium ions were combined with bentonite, where the former were used as adsorbate and the latter as adsorbent, respectively. We characterized pre- and post-adsorption states of adsorbent by using terahertz techniques. A detailed mechanism of the adsorption of trivalent chromium on the prepared adsorbent was explained based on the obtained results of adsorption investigations.

Various methods for detecting heavy metal ions have emerged in recent years^29^. The commonly used techniques are X-ray diffraction (XRD)^30^, X-ray fluorescence spectrometry^31,32^, surface enhanced Raman scattering (SERS) and Raman techniques^33^, infrared spectroscopy^34^, reflection spectroscopy, etc. However, all these methods have a number of drawbacks. In particular, they are more expensive and time-consuming as compared to terahertz technology. The measurement process using the latter technology is very easy and does not require the addition of highly expensive reagents or creation of complex measurement conditions. Moreover, these methods are available only in high-end laboratories. Furthermore, sample preparation also requires much effort. Therefore, use of terahertz time-domain spectroscopy is a suitable alternative to overcome these shortcomings.

Terahertz waves are usually defined as far-infrared electromagnetic radiation^35^ with frequencies in the range of 0.1 to 10 THz^36–38^ corresponding to the wavelengths between 30 µm and 3 mm. They belong to a specific wavelength band between micro- and infrared waves. Due to the specificity of the electromagnetic spectrum of terahertz waves, their interaction with materials has unique physical properties. Terahertz technology is widely used for safety applications^39^ as well as for qualitative and quantitative detection of drugs, solutions, and characterization of material quality^40,41^. Terahertz technology has many advantages as compared to other medical diagnosis methods^42^. Terahertz radiation does not cause ionization in biological tissues due to very low photon energy^43^. Terahertz technology can be used for imaging spatial distribution and penetration of drug application sites^44^. This technology has been also applied to detect agricultural pollution^45^. Terahertz techniques offer significant advantages for qualitative and quantitative studies of heavy metal ions in soil contaminants because of the complex structure of soil and its unique absorption properties for terahertz waves^46–48^.

## Results

### Terahertz time-domain spectroscopy

**Figure 1:**
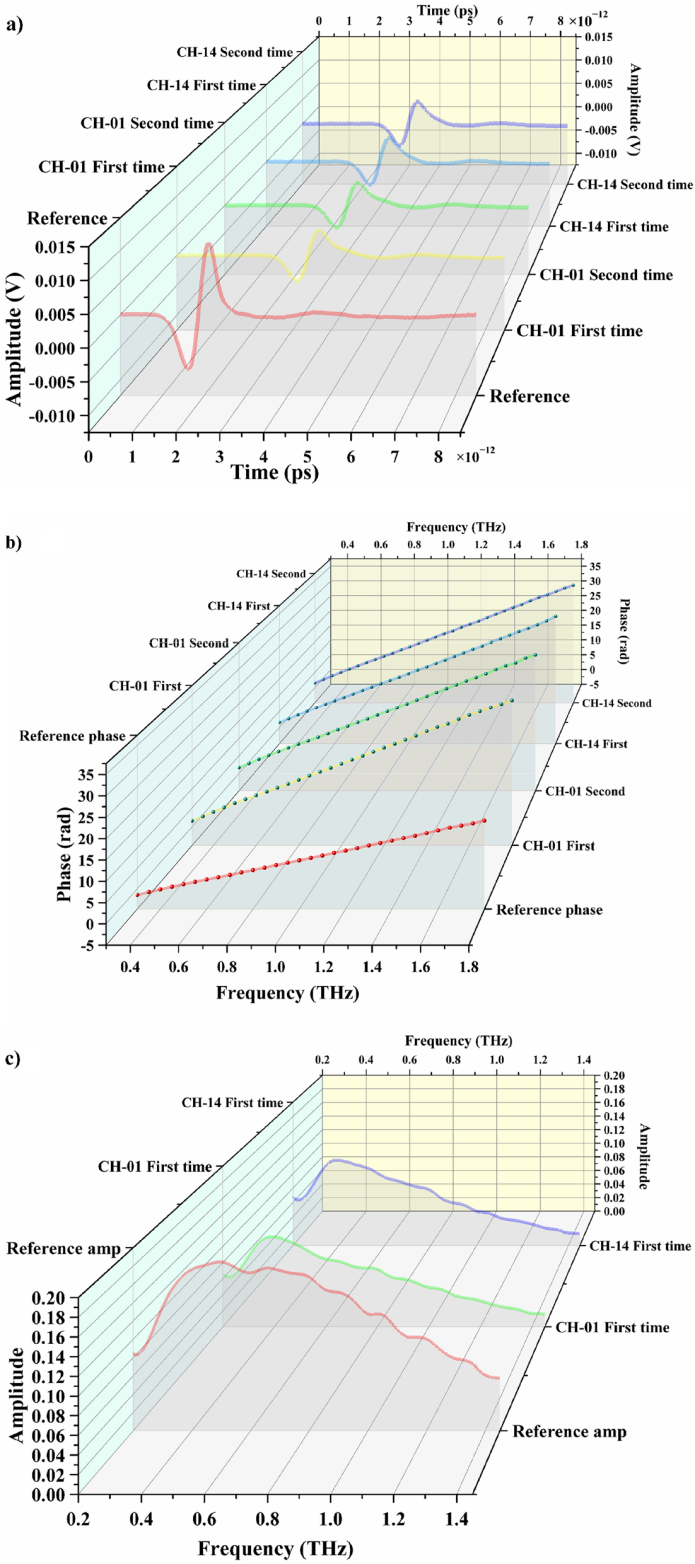
Results of terahertz measurements on raw bentonite samples. Terahertz time-domain spectra (**a**). Phase diagrams for two sets of raw bentonite samples (**b**). Frequency-domain spectra (**c**). The experiments were carried out at the temperature of 293.15–298.15 K, the pressure of one atmosphere and the humidity of air inside the setup < 3.5%.

Terahertz time-domain spectra (Fig. 1a) of the bentonite samples (CH-1 and CH-14) were obtained by terahertz measurements at the same weight values. Figure 1b,c are derived from Fig. 1a using the principle of partial algorithm. In particular, Fig. 1b shows phase diagrams for two different equal-quality sets of samples, which entirely overlap. Figure 1c shows that the amplitude charts for two groups of the original bentonite samples of equal quality obtained from terahertz measurements coincide. These results demonstrate the similarity of the petrographic characteristics of the bentonite samples and confirm that terahertz measurements can be effectively used to identify the material and obtain its qualitative characteristics.

**Figure 2:**
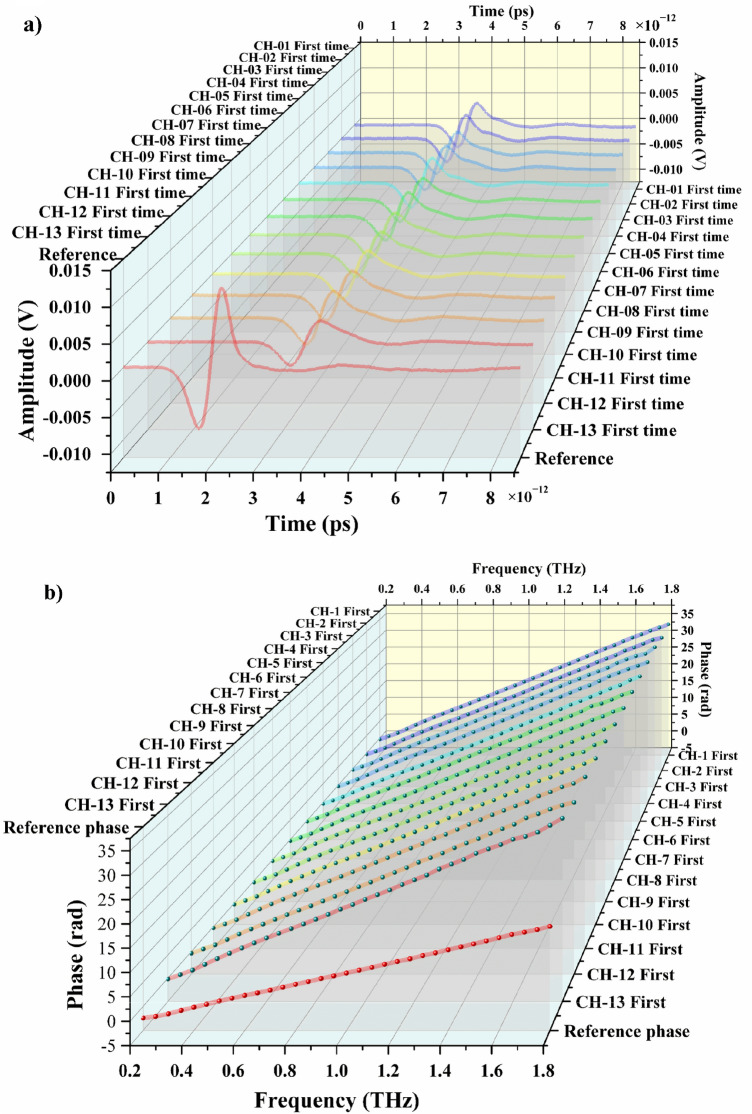

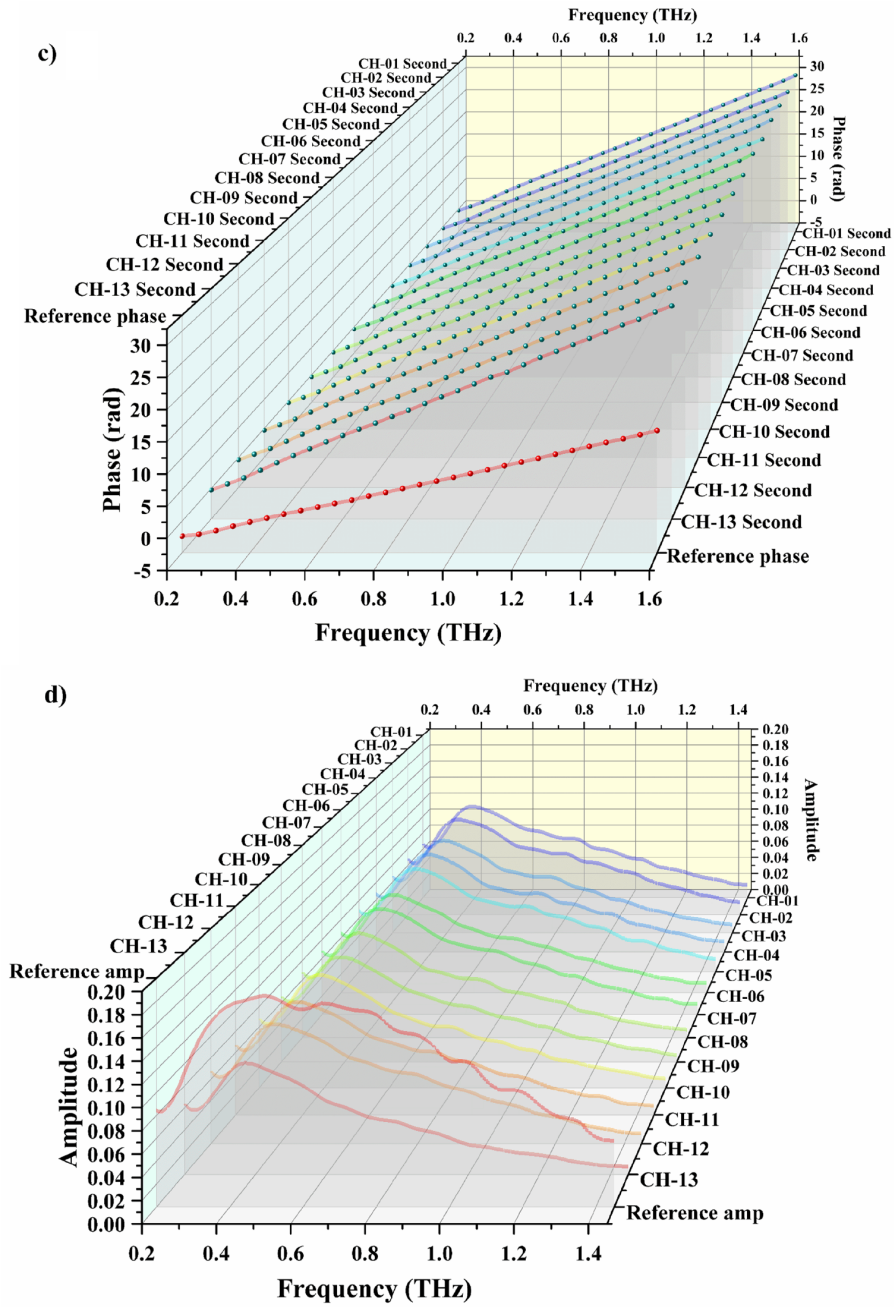
Results of terahertz measurements on bentonite samples. THz-TDS diagrams (**a**). THz-TDS phase diagrams obtained in the first test of all samples (**b**). THz-TDS phase diagrams obtained in the second test of all samples (**c**). Frequency-domain spectra obtained in the absorption by bentonite samples with different weights at different concentrations of trivalent chromium ions (**d**). The experiments were carried out at the temperature of 293.15–298.15 K, the pressure of one atmosphere and the humidity of air inside the setup < 3.5%.

The bentonite sample CH-1 (Fig. 2a) was tested at the weight of 1.5, 2.0, 2.5, and 3.0 g. Terahertz measurements were carried out after absorption of 0.5, 1.0 and 2.0 mol/L chromium ion solution. The results obtained after data processing are presented by the frequency-domain spectra (Fig. 2d) and the spectral phase diagrams (Fig. 2b,c). The phase diagrams for two measurements overlapped when the measurements were performed on the same type of bentonite before and after absorption. This indicates that the structure of bentonite does not change significantly due to the absorption of chromium ions.

However, terahertz analysis in the high- and low-frequency ranges is not reliable. The absorption coefficient of water, where the test samples are stored all the time, increases with the frequency of terahertz radiation. Therefore, the high-frequency signal is weaker when the terahertz radiation passes through thick samples. In its turn, the signal in the low-frequency range is mainly affected by the setup noise. Thus, a suitable range of frequencies for analysis should be carefully chosen.

**Figure 3:**
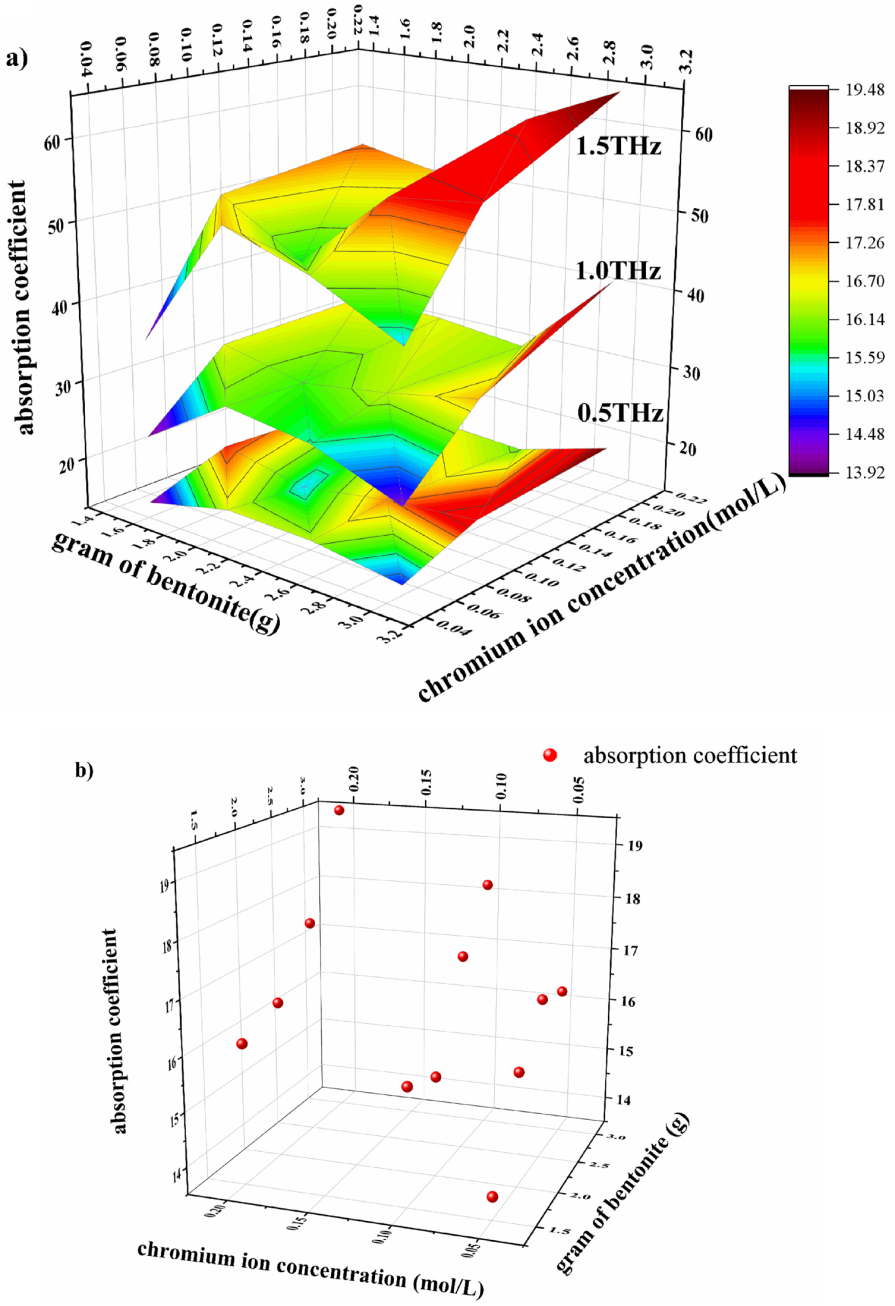
Results of terahertz absorption measurements on bentonite samples at different frequencies. Diagram of absorption relationship at the frequency of 0.5, 1, and 1.5 THz (**a**). Absorption coefficient versus bentonite weight and chromium ion concentration at the frequency of 0.5 THz (**b**). The experiments were carried out at the temperature of 293.15–298.15 K, the pressure of one atmosphere and the humidity of air inside the setup < 3.5%.

The amplitude characteristics of the sample as a whole resulted from the absorption of chromium ions are clearly seen from the absorption map for the first THz-TDS test (Fig. 2d). The values of absorption coefficient at 0.5, 1 and 1.5 THz were selected by analyzing the data presented in Fig. 2d to build Fig. 3a. Taking into account the relationship between the amplitude at certain weight of bentonite and the concentration of chromium ions, it is evident that the CH-2 sample point is on top and the CH-13 sample point is at the bottom of all the sample points. The relationship between the amplitude values and the values of absorption coefficient presumably exists (Eqs. (1) and (2). Therefore, we inferred the amplitude values as a function of the absorption values.

Figure 3a shows the dependence of absorption coefficient on the weight of bentonite sample and the concentration of chromium ions in the solution. The general trend is that the absorption coefficients measured at all the frequencies decrease in the direction of low chromium ion concentrations and low bentonite weights. The absorption rate increases with the weight of bentonite and the concentration of solution. Moreover, there is a significant difference between the values of absorption coefficient corresponding to different terahertz wave frequencies and the same concentrations of chromium ions and bentonite weights. Observed phenomena support the suitability of the quantitative detection of substances by terahertz measurements.

The rate of absorption increased with the increase of chromium ion concentration and weight of bentonite sample. The value of absorption coefficient at 0.5 THz was calculated by Eqs. (1) and (2) as shown in Fig. 3b. The values of absorption coefficients obtained in the THz-TDS tests for the samples from CH-02 to CH-13 were as follows: 13.925, 15.620, 16.518, 16.277, 15.670, 15.358, 17.178, 18.215, 16.088, 16.416, 17.557, and 19.463. Slope 1, slope 2 and slope 3 were obtained by linear regression fitting without maintaining strict accuracy using generalized estimating equations. These slopes refer to the ones of the rate of changing absorption coefficient obtained at the increase of the weight of bentonite sample (1.5–3 g) at the concentrations of chromium ions of 0.05, 0.1 and 0.2 mol/L, respectively. They are equal to 0.795, 0.946 and 1.127, respectively, which are positive values. It is evident that the absorption coefficient increases with the increase of bentonite weight at fixed values of initial chromium ion concentration. Furthermore, the absorption coefficient shows a significant increase with the increase of chromium ion concentration at fixed values of the weight of bentonite. Therefore, the absorption process on bentonite has a positive feedback characteristic, so that the absorption efficiency is improved with the increase of bentonite weight. Such characteristic is caused by that the absorption of chromium ions by montmorillonite.

We calculated the dependence of the slope of absorption coefficient on the serial number of bentonite samples. Increasing from linear regression fit using generalized estimating equations and the Slope is 0.301. Slope refer to the ones of the rate of changing absorption coefficient obtained with the increase of the serial numbers of samples. The serial numbers of samples corresponded to numbered sections 2 to 13, i. e., the serial number of CH-02 sample was 2 etc. It can be concluded that the value of absorption coefficient increases with the increase of the weight of bentonite sample and the concentration of chromium ions and also that terahertz can be used for quantitative analysis within a scalar.

### Spectrophotometry

**Figure 4:**
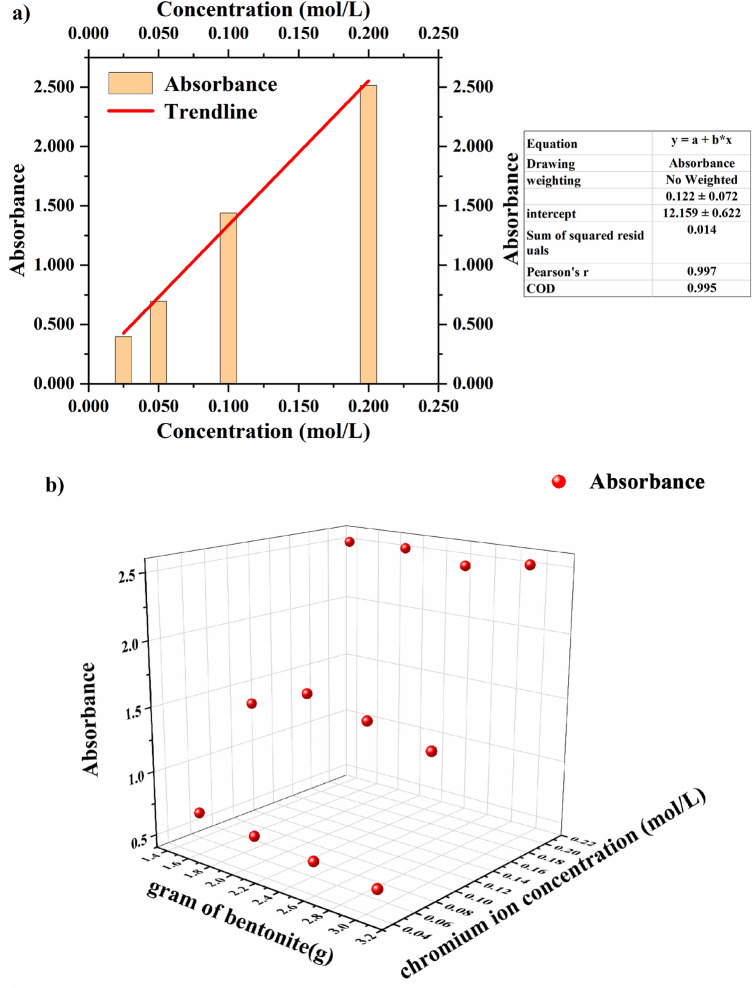
Diagram of the relationship between the absorbance of standard sample and chromium ion concentration (**a**). Yellow rectangles represents the sample and red straight line is the standard curve. Fitting equation and its parameters are provided in the Figure. Diagram of the relation between the absorbance, the chromium ion concentration and the weight of bentonite (**b**). Red spheres correspond to the values for absorbance of different samples. The experiments were carried out at the temperature of 293.15–298.15 K and the pressure of one atmosphere.

Results of spectrophotometry show that the absorbance linearly depends on the concentration of chromium ions in the solution (Fig. 4a). The solution concentration is obtained when the adsorption process is completed and the solution is centrifuged. The absorbance of the solution after absorption drops linearly with the increase of the amount of bentonite in it. This linear dependence is caused by the inverse relationship between the solution and bentonite absorption.

Initially, the concentration of chromium ions was constant and had the values of 0.05 mol/L, 0.1 mol/L, and 0.2 mol/L. The linear dependence of absorbance on the solution concentration was analyzed according to the Beer–Lambert law. The slope of the equation in Fig. 4a range from 11.537 to 12.781 (slope ± SD, 12.159 ± 0.622), and the y-intercept ± SD of is 0.122 ± 0.072. The linear fit of the absorbance of standard sample versus chromium ion concentration was excellent with the goodness of fit index equal to 0.997.

The results of analysis for constant weight of bentonite are shown in Fig. 4b. The values of absorbance for the samples from CH-02 to CH-13 presented in this figure are as follows: 0.666, 0.627, 0.587, 0.545, 1.398, 1.581, 1.482, 1.377, 2.515, 2.523, 2.450, and 2.523. Slope 1, slope 2, and slope 3 (they are not indicated in the figure) were generated by linear regression fitting without maintaining strict accuracy using generalized estimating equations. Slopes 1, 2 and 3 refer to the ones of the absorption coefficient obtained at the increase of bentonite weight (1.5–3 g) at the concentrations of chromium ions of 0.05, 0.1 and 0.2 mol/L, respectively. These slopes are equal to − 0.365, − 2.732 and 0.119, respectively. The fluctuation of the value of slope 3 is around 2.500. Therefore, this slope is not used for the calculations due to the limitation of the maximum measurement range of spectrophotometer. Both slope 1 and 2 are negative. It is evident that the absorbance linearly depends on the weight of bentonite at constant solution concentration. On the other hand, the serial numbers of samples gradually increase, which means that the absorbance linearly changes with the chromium ion concentration.

Because of these two relationships that two values represented by slope 1 and slope 2, absorbance is a binary function of the concentration of trivalent chromium ions and the weight of bentonite. The relationship between the absorbance and the chromium ion concentration is expressed by the Beer–Lambert law. The absorbance of solution is also a function of its concentration, which means that the absorbance of a solution is a function of the absorption of bentonite. As a result, the latter is a binary function of the concentration of trivalent chromium ions and the weight of bentonite. The adsorption process obeys a quasi-second-order kinetic model.

### XRD tests

**Figure 5:**
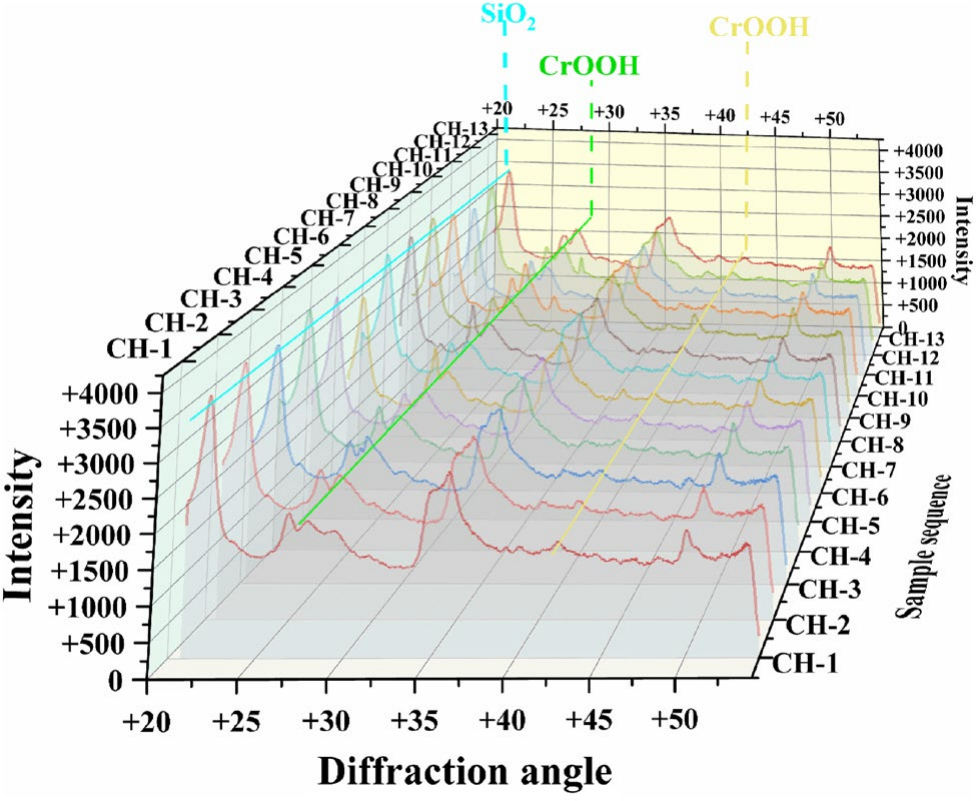
XRD diffractogram of bentonite with adsorbed chromium ions. Different colors of curves correspond to different samples. The wave peaks and troughs correspond to different diffraction angles. The experiments were carried out at the temperature of 293.15–298.15 K and the pressure of one atmosphere.

Diffraction of X-rays on crystals enables to register X-ray diffraction spectra. Analysis of such spectra provides the information about the crystal material. The XRD diffractogram is shown in Fig. 5. It can be seen from this figure that this curve remains unchanged after the adsorption process. CH-01 was a reference bentonite sample, while the samples from CH-02 to CH-13 were subjected to XRD measurements after the dynamic adsoption experiment. No change of intensity of from bentonite samples CH-01 to CH-13 was observed at the diffraction angle 2θ = 21.984°. This apparently demonstrates that the inter-layer structure of bentonite was preserved during the adsorption process. In particular, the diffraction angle for SiO_2_, being the main component of bentonite clay structure, is 2θ = 21.984°. The XRD results are compatible therefore with the results obtained in terahertz measurements and confirm that the structure of bentonite does not significantly change due to the adsoption of chromium ions.

XRD can be also used to determine the values of lattice parameters of materials and to study structural changes in a semi-quantitative manner. The diffraction peaks at 27.681° ((110) plane) and 42.717° ((210) plane) are the main diffraction peaks of CrOOH. The adsorption of chromium ions is known to occur, but the overall adsorption is modest. It is also worth to note that the amount of adsorbed substance cannot be reliably quantified due to the semi-quantitative results provided by XRD. Estimation of the concentration of a substance from XRD results is based on the RIR or K of the card. Hence, the estimated values can contain great errors. Usually, the error of the estimation of concentration based on XRD results is around 10%. This is the reason for our inability to perform precise quantification by using XRD method.

### ICP-MS tests

**Figure 6:**
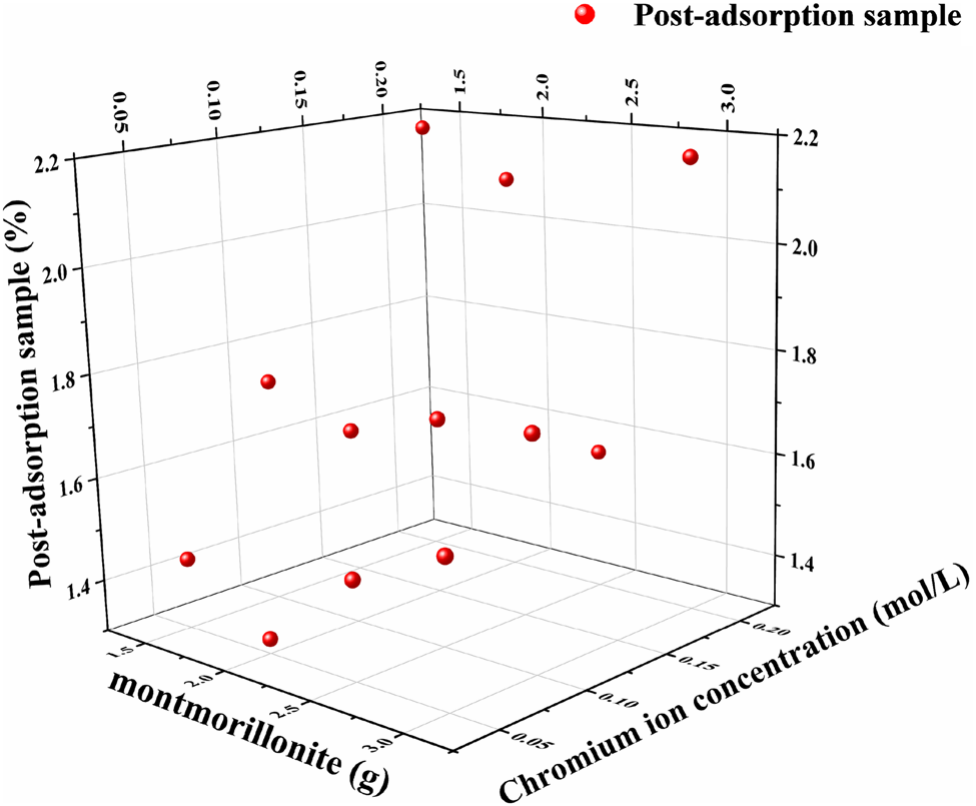
Distribution of the concentration of chromium ions in the samples after absorption. Red spheres correspond to different samples. The experiments were carried out at the temperature of 293.15–298.15 K and the pressure of one atmosphere.

Results of the ICP-MS analysis of samples after adsorption are shown in Fig. 6. The residual concentrations of chromate ions in post-absorption samples from CH-02 to CH-13 presented in Fig. 6 are as follows: 1.436, 1.328, 1.490, 1.579, 1.736, 1.674, 1.732, 1.742, 2.179, 2.090, 1.572, and 2.165%. The absorption values of the ICP-MS tests are the adsorption efficiencies of bentonite. Slope was generated by linear regression fitting without maintaining strict accuracy using generalized estimating equations. The slope refer to the ones of the rate of changing adsorption coefficient obtained at the increase of serial numbers of samples. The adsorption coefficient increased with the increase of the serial numbers of bentonite samples and the slope amounted to 0.061.The slope is a positive value. The intensity of signal corresponding to chromium ions retained in bentonite after adsorption increased significantly with the increase of chromium ion concentration (Fig. 6). This means that increase of the concentration of chromium ions due to adsorption leads to the increase of bentonite absorption capability. The concentration of solution decreased because bentonite adsorbed trivalent chromium ions from it. The ICP-MS data showed roughly the opposite trend to the one obtained based on the measured spectrophotometry data for solution.

The ICP-MS results were identical to the results of terahertz measurements, demonstrating that the latter may be used for quantitative sample analysis. Output data from the THz-TDS analysis were normalized to the ICP-MS values for the control samples. The overall adsorption efficiency of bentonite calculated in this experiment was around 1%. The efficiency of adsorption raised up to around 2.1% with the increase of bentonite mass or chromium ion concentration.

Both ICP-MS and terahertz tests showed absorption of chromium ions by bentonite, which is influenced by the concentration of chromium ions and the weight of the bentonite with linear relation. The absorption efficiency improves with the increase of the weight of bentonite and the concentration of chromium ions.

## Discussion

In this study, adsorption capacity of bentonite is characterized by the results of terahertz absorption measurements. It is quite evident that this method offers a means for detecting the concentrations of heavy metal pollutants. The THz-TDS results were further validated using spectrophotometry, XRD, and ICP-MS. In our study, THz-TDS served as a method for qualitative and semi-quantitative analysis of materials. THz-TDS is holistic, safe, and straightforward. The most prominent is that the permselectivity of THz-TDS has an important influence on its results. THz-TDS shows a high degree of differentiation of non-metallic and non-polar substances due to accurate identification of molecular and chemical bonds. This makes it different from the ICP-MS method, which is superior in atoms. Hence, THz-TDS is a better choice for qualitative and semi-quantitative testing of non-metallic and non-polar substances as compared to ICP-MS when the accuracy requirements are not stringent.

The terahertz amplitude of bentonite has a maximum at the frequencies less than 0.5 THz. It was also observed that the character of the increase of adsorption rate can be changed by tuning the weight of bentonite and chromium concentration. The reliability of THz-TDS measurements was assessed by dividing the THz-TDS results by the corresponding ICP-MS ones. Obtained ratios for the samples from CH-02 to CH-13 are as follows: 0.103, 0.085, 0.090, 0.097, 0.111, 0.109, 0.101, 0.096, 0.135, 0.127, 0.090, and 0.111. The estimated values were obtained by linear regression fitting without maintaining strict accuracy using generalized estimating equations and the slope value is 0.0018. We supposed that the relation between the THz-TDS results and the ICP-MS ones was the constant-coefficient equation at too small values of slope. This points to that terahertz technique may be applied for quantitative analysis of materials but requires a reference standard for this. This issue will be considered in our future studies.

Finally, using THz-TDS, we made a quantitative study of the sorption of chromium ions from solutions of different concentrations by bentonite samples of different weights. Based on the relationship between the ICP-MS test results and the THz-TDS ones, we calculated the adsorption coefficient of bentonite to be 1.44% at the concentration of chromium ions of 0.05 mol/L and the weight of bentonite sample involved in the adsorption of 1.5 g. The adsorption rate of chromium ions by bentonite significantly increased and the value of adsorption coefficient could reach about 2.1% with the increase of chromium ion concentration and the mass of bentonite involved in the absorption.

In this work, terahertz time-domain spectroscopy was used to qualitatively and semi-quantitatively characterize adsorption of trivalent chromium ions from solutions by bentonite. The advantages of THz-TDS are low energy as well as non-destructive, fast and damage-free measurement process. Using the proposed method, the relationship between the adsorption coefficient, concentration of chromium ion solutions and bentonite weight were successfully measured and characterized. Besides, we calculated the efficiency of the adsorption of trivalent chromium ions by bentonite. The terahertz technology may be considered promising to meet the ever-increasing requirements in mineral analyses for rapid detection of chemical contaminants.

## Methods

### Principle

#### Spectrophotometry

Chemical substances absorb or reflect light in specific wavelength ranges. In a spectrophotometer, light passes through the sample under study. The intensity of transmitted light is measured to estimate the intensity of the light absorbed by a substance to analyze its chemical composition or concentration of known elements^49^. Transition of electrons takes place between different levels depending on the wavelength of light passing through the material. Since different wavelengths of light mean different photon energies, the light absorption rate is wavelength-dependent.

The absorption wavelength of 600 nm was used to determine the presence of chromium ions by a 617 ultraviolet spectrophotometer (722S, Shanghai Precision Scientific Instrument Co., Ltd, Shanghai, China). The spectrophotometer was pre-heated for 30 min before the measurements and its wavelength was adjusted to 600 nm. Containers for spectrophotometer cuvettes were triple rinses with chromium ionic liquids with the concentrations of 0.025, 0.05, 0.1 and 0.2 mol/L. After this, chromium ionic liquids were pipetted into the cuvettes.

The excess solution around the cuvettes was wiped off with absorbent paper. The cuvettes were placed into the test tank, and the sample chamber was covered. The performance of measurement device is correct if 100.0 is displayed when the transmittance button 100% is pressed after closing the cover and 0 is displayed if the 0 button is pressed after opening the cover. Then the device cover was open and the instrument changed to the patterns of absorbance. At this, 0.00 was displayed on the screen. The cuvette was placed in the light pathway by pulling a control lever. The measurement data, namely the absorbance of solution in the cuvette No. 1, were recorded on data sheets. This sequence of operations was repeated four times for each sample in order to obtain mean values. A total number of 64 measurements were carried out in this experiment.

The spectrophotometer can only perform measurements on solutions. Therefore, obtained measurement results refer to a liquid environment. On the other hand, the results of THz-TDS are obtained for solids. In this respect, spectrophotometry enables to obtain a side view of the terahertz measurement results and to provide a more comprehensive explanation of the process of heavy metal adsorption by bentonite.

#### Terahertz time-domain spectroscopy

**Figure 7:**
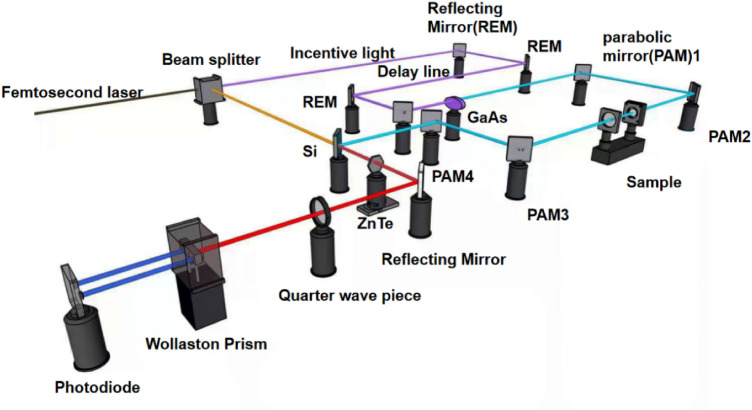
Schematic diagram of the transmission THz-TDS system. Incident and transmitted terahertz waves are shown with blue lines.

A transmission THz-TDS system was used in this study. This system consists of a femtosecond laser, a photoconductive antenna that generates terahertz radiation, a detection system and a time-delay control system. The schematic view of the experimental setup is shown in Fig. 7.

Femtosecond laser light was generated by a laser diode driven by a femtosecond laser (green line in Fig. 7). This light was divided by a beam splitter into two beams. One beam acted as a probe light (yellow line), and the other one (purple line) was focused on the GaAs photoconductive antenna^50,51^ as a pump light to generate terahertz pulses^52^. Generation of terahertz radiation is based on photoconductivity. This is a resonant process in which a femtosecond optical pulse is absorbed by GaAs through interband transitions with generation of free charge carriers. These carriers are accelerated either by an externally applied voltage or by a built-in electric field in the depletion or accumulation region of the semiconductor. A transient current is generated in this way, which induces emission of terahertz electromagnetic radiation.

Parabolic mirrors (PAM1 and PAM2) were used to focus the beam on the sample under study. Terahertz beam passing through the sample is directed toward the detector by PAM3 and PAM4. Both the terahertz pulses and the probe light beam pass through the ZnTe electro-optical crystal. The electric field of terahertz waves causes an electro-optical effect in the electro-optical crystal changing its refractive index. In its turn, change of refractive index can induce a change of the polarization state of linearly polarized probe light. The optical signal is photoelectrically converted to the electrical one. This beam is split by a Wollaston prism and received by a balanced photodiode. A critical factor of the THz-TDS system is the coupling between the emitter and the detector. Lenses made of silicon (Si)^53^ or other high-index materials are often used in THz-TDS systems to ensure high efficiency of such coupling. Moreover, parabolic mirrors are used to guide terahertz radiation in free space from the emitter to the detector. A system consisting of two pairs of parabolic mirrors arranged to have the emitter, detector and the midpoint in confocal planes, is often used in terahertz imaging and optical pump-THz probe systems. To reduce the absorption of terahertz radiation by water vapor, spectrometer is often purged with dry air or nitrogen gas^54^.

During the experiments, THz-TDS setup obtains a time-domain signal by detecting the sample or a reference signal in the absence of sample in the setup. Fourier transformations are used to convert the time-domain signal into amplitude and phase of the sample in the frequency domain. By comparing the amplitude and the phase of the signal from the sample and the reference one, the refractive index and absorption coefficient are calculated by the following expressions:

1 $$n = \frac{c\varphi }{{2\pi d\omega + 1}}$$

2 $$\alpha = \frac{4\pi k\omega }{c} = \frac{2}{d} \times \ln \left[ {\frac{4n}{{A\left( {n + 1} \right)^{2} }}} \right]$$

where c is the speed of light, φ is the bit-phase difference between the signal from the sample and the reference one, d is the sample thickness, ω is the frequency, k is the wave vector, and A is the amplitude, respectively.

To measure terahertz signal, the THz-TDS system was used in the reflection mode. GaAs photoconductive antennas were driven by a femtosecond fiber laser to generate and detect terahertz pulses. The spectral range was from 0.1 to 3.5 THz. The spectral resolution of the terahertz comb spectroscopy system was set to 1 GHz to match it to the pressure-induced broadening of absorption linewidth with a temporal resolution of less than 1 ps. The infrared femtosecond laser operated at the wavelength of 800 nm. Requisite electric field was achieved with ultrashort pulses of the width of less than 100 fs.

The THz-TDS device used in this study was Terahertz Air Biased Coherent Detection (CIP-ABCD, Daheng New Epoch Technology, Inc.). The measurements were carried out after completion of adsorption process. All samples were prepared in powdered form with a fixed weight of 0.2020 g by mixing with polytetrafluoroethylene (PTFE) at a mass ratio of PTFE to bentonite of 3:7. The pooled samples of about 0.2020 g were weighed after stirring for 20 min, pressed for two minutes at 4 mpa and then tested by THz-TDS.

Sample testing started only after the air humidity dropped below 3.5%. The test was repeated two times for each sample in order to obtain mean values. A total number of 42 tests were performed in the terahertz time-domain spectroscopy experiment involving 12 groups of samples after the adsorption process and 2 groups of reference samples.

#### X-ray diffraction

X-ray diffraction is a powerful tool for detection of the crystallinity of materials under study. In this work, XRD was used to analyze the amorphous versus the crystalline structure of our samples. Moreover, the diffraction spectra also enable to reveal the differences in the elemental composition of samples.

The intensity of diffraction (I) and the spacing between the lattice planes in the crystal (d) are two main characteristics of an X-ray diffraction spectrum. I refers to the type, number, and positions of diffraction peaks, while d is a key characteristic of crystal cells that describes the cell size and shape. XRD pattern of a crystal is unique in terms of interlayer spacing and diffraction intensity. Therefore, XRD is an excellent technique to identify substances. Comparison of XRD spectra obtained in the experiment with the standard data provided by the Joint Committee on Powder Diffraction Standards (JCPDS) enables to retrieve the substance composition.

The XRD device used in this study was D8 discover XRD. 13 groups of bentonite samples were tested using X-ray diffraction, each group being tested twice. The XRD analysis aimed to determine structural changes resulted from the adsorption. Moreover, it can be used as a standard method to validate the results of the terahertz measurements of bentonite structure.

#### Inductively coupled plasma mass spectrometry (ICP-MS)

Inductively coupled plasma mass spectrometry is an emergent technique for detection, characterization and quantification of nanoparticles. Inductively coupled plasma (ICP) is an ionization source, and it can create high-temperature plasma by an electromagnetic coil that applies high-power, higher frequency signals and push inert gas through the sample. The mixed gas of the sample and inert gas is pushed to keep the ionized body equilibrium and continual ionization after a succession of evaporation, decomposition, excitation, and ionization procedures in the core zone. High-temperature plasma primarily ionizes material producing positive monovalent ions and electrons. Ions in the plasma are separated and delivered to the mass spectrometer via the ICP-MS interface, where the receiver target receives them. The quality screening and analysis of mass spectrometer and detection of ions with varied nuclei to mass ratios make the associated ions recognized and determine their elemental strengths. The possibility of the detection of elements in both solutions and particles makes this method very promising in nanoscience research.

In this experiment, the ions of the samples were quantified using an inductively coupled plasma mass spectrometer (ICP-MS, Agilent 7500 cx). 13 groups of bentonite samples were tested using X-ray diffraction. One group was tested at a time. This enabled to carry out quantitative measurements of bentonite samples by ICP-MS with high accuracy. In our work, the ICP-MS and THz-TDS measurements were performed on solid samples. ICP-MS results confirmed the results of the terahertz measurements of chromium concentration, which provides a strong support of THz-TDS for quantitative detection of heavy metal ions.

#### Data processing methods

Beer–Lambert law describes the relationship between the intensity of light absorbed by a substance at a particular wavelength, the concentration of absorbing substance and the thickness of its liquid layer^55^. In this study, the temperature, pressure, sheet thickness and other parameters were controlled to ensure proportionality of sample concentration to the ideal absorbance. We started to investigate the relationship between the bentonite weight, chromium ion concentration and adsorption using the Beer–Lambert law.

### Experimental workflow

**Figure 8:**
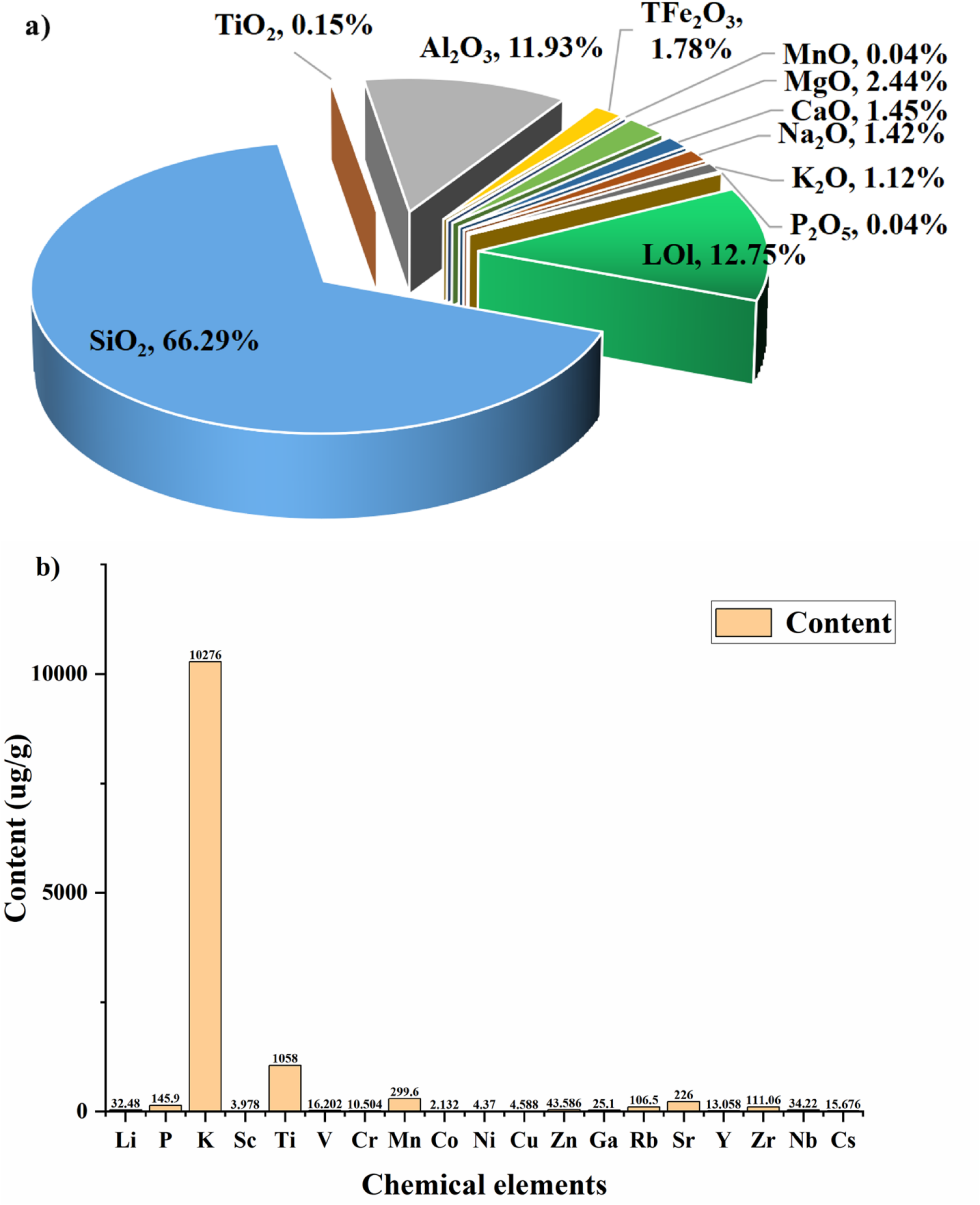
Pie chart of the chemical composition of primitive bentonite samples (**a**). Blue, green and gray colors represent SiO_2_, loss-on-ignition (LOI) and Al_2_O_3_. Histogram of the content of trace elements in primitive bentonite samples (**b**). K and Ti have the highest contents. The experiments were carried out at the temperature of 293.15–298.15 K and the pressure of one atmosphere.

In this study, bentonite powder was prepared by grinding solid gray colored bentonite samples. The contents of aluminum trioxide (Al_2_O_3_) and silicon dioxide (SiO_2_) in the samples were measured by inductively coupled plasma optical emission spectroscopy (ICP-OES) (China University of Geosciences (Beijing) Research Institute). The measured contents of these substances are presented in Fig. 8a. Na (2.84%), Ca (1.45%) and K (2.24%) elements were also detected. These elements are stable and not involved in reactions. The montmorillonite, which was the most abundant mineral in bentonite, mainly consisted of Al_2_O_3_ and SiO_2_. Hence, the contents of the two mentioned compounds determined the grade of bentonite. The content of SiO_2_ dust was 66.29% and the content of Al_2_O_3_ dust was 11.93%. To avoid the influence of loss-on-ignition, the content of rocks was reconverted. The internal content of silica was found to be 75.98% and the content of alumina trioxide was 13.67%. The sum of these two numbers make 89.65%, which indicates that the samples were clay-like and could be used as the samples in this study.

40 mg sample weighed on automatic electronic balance was placed in a mixture of two acids (HNO_3_ + HF) to chemically pre-process the material. The trace elements present in this sample were determined using Agilent 7500 plasma mass spectrometer. Standard solutions labelled as STD-1, STD-2 and STD-4, provided by the American Bureau of Standards Equipment Laboratory (ABSEL), were used as the external standard. Moreover, Rh was added as the internal standard for calibration. In our experiments, standard specimens such as AGV2, BHVO-2, and W-2 provided by the United States Geological Survey (USGS) as well as full-flow dissolved rock samples such as GSR-1 and GSR-3 provided by China Geological Testing Center (CGTC) were used as standard samples for comparative analysis.

The content of chromium ions in the mineral samples was determined (Fig. 8b) to check their purity. The contents of other ions were also found to exclude the errors introduced by them. It was established that K, Mn, P and Zr were the most abundant elements among the total number of 19 inorganic trace elements found in bentonite, and the content of Cr was 10.504 ug/g. The distribution of elements was close to the average distribution of elements in the upper part of the Earth's crust. This indicates that the samples used in the experiment were natural bentonite. In principle, chromium ions filling each reservoir of bentonite do not react with other ions. Cr^3+^ binds to OH^−^ of inter-layer H_2_O molecules in bentonite to form CrOOH, Cr(OH)_3_ and other compounds. The adsorption of chromium ions by bentonite took place according to the following reaction:

3 $${\text{Cr}}^{3 + } + \left( {{\text{Al}}_{2} ,{\text{Mg}}_{3} } \right){\text{Si}}_{4} {\text{O}}_{10} {\text{OH}}_{2} \cdot {\text{nH}}_{2} {\text{O}} \to {\text{CrOOH + Cr}}\left( {{\text{OH}}} \right)_{3} + \cdots$$

For sample preparation, 1.5, 2.0, 2.5 and 3.0 g of bentonite were placed in a shaker and shaken at a constant temperature of 302.15 K for 3 days with a minimum speed of 250 rpm/min. After that, the shaker was turned off and the samples were stored for 1 day. Finally, the samples were centrifuged for 10 min at 10,000 rpm/min. After centrifugation, the supernatant in the centrifuge tube was transferred to a new centrifuge tube. The ions on the surfaces of remaining solids were washed by deionized water in order to remove chromium ions adhering to the solid surface after. The remaining solid samples were placed in an evaporation dish in an oven and dried at 331.15 K during 24 h. After being dried, the samples were grounded to about 200 mesh for subsequent study.

**Figure 9:**
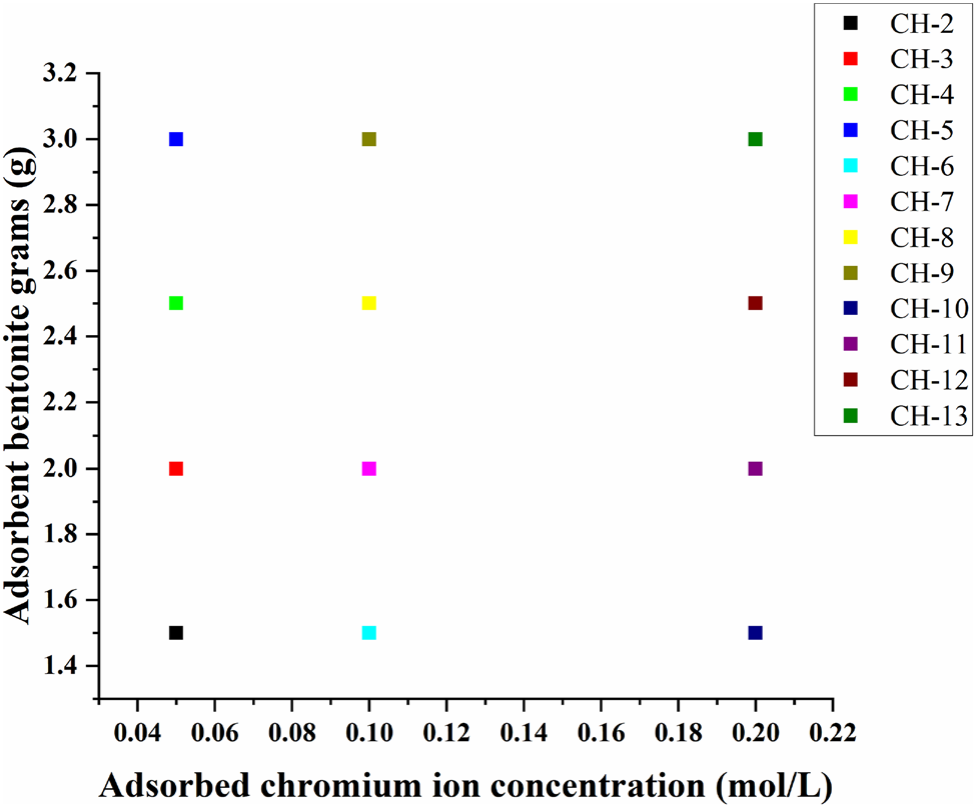
Diagram of the relationship between the weight of bentonite samples and the concentration of the adsorption solution of trivalent chromium ions for different sample numbers.

Then deionized water was added to bentonite to get adsorb the blank group. The synthesized bentonite was labelled as CH-1 and commercial bentonite was labelled as CH-14. The relationship between the serial number of sample and the weight of bentonite as well as the chromium ion concentration is shown in Fig. 9. It clearly seen from this figure that the serial number increases with the increase of bentonite weight and the concentration of chromium ions.

#### Analysis of solid samples

After the separation of solid from liquid the solid surface was rinsed with a small amount of deionized water to wash off the residual solution and dried in an oven at a constant temperature of 333.15 K for 2 days. After this, a solid powder of about 200 mesh was prepared by grinding. A portion of the sample was added to the XRD sample solution and left for one hour. It was finally tested using a Bruker D8 DISCO device. The sample was placed in the XRD sample bath and kept there during one hour. One part of the sample was subjected to ICP-MS measurements to determine the amount of trivalent chromium ions. The other part was mixed with PTFE at a ratio of 3:7 during 15 min. The 0.2 g portion of the sample was pressed at 4 Mpa for 2 min to produce a test sample for THz-TDS. The results of XRD measurements were compared to the results obtained by THz-TDS.

#### Analysis of liquid samples

Five milliliters of the solution were poured in the 50 ml cuvette with a pipette gun. The absorbance was measured at 600 nm using a 617 spectrophotometer to determine the concentration of chromium ions. Finally, the relationship between the change of the concentration of adsorped ions, the bentonite content and the concentration of chromium ions configured was determined. The adsorption process of chromium ions by bentonite can be better presented by solid-liquid comparison of the results obtained by THz-TDS, ICP-MS, XRD, and spectrophotometry.

## Data Availability

Data underlying the results presented in this paper are not publicly available at this time but may be obtained from the authors upon reasonable request.
